# Comments on “Biofilms of *Candida albicans* serotypes A and B differ in their sensitivity to photodynamic therapy”

**DOI:** 10.1007/s10103-014-1591-7

**Published:** 2014-05-27

**Authors:** Mariusz Grinholc

**Affiliations:** Laboratory of Molecular Diagnostics, Department of Biotechnology, Intercollegiate Faculty of Biotechnology, University of Gdansk and Medical University of Gdansk, Kladki 24, 80-822 Gdansk, Poland

Dear Editor,

In the April 2014 issue of the online-first article, Rossoni et al. [1] reported that *Candida albicans* serotype B is more sensitive to photodynamic treatment than serotype A.

As we know, significant variation among particular strains within the same species in response to the photodynamic treatment exists [2]. The differences can reach several logs in survival of viable counts. We have previously shown that taking into consideration the genetic background of microorganisms, i.e., *Staphylococcus aureus*, within the same closely related strains, assigned as a single epidemic clone, we can identify strains that are highly resistant as well as highly sensitive to photodynamic inactivation (PDI) [3]. Thus, just having a sufficiently large database of strains, one can properly infer about the importance of a particular feature in response to PDI. Unfortunately, as far as I am concerned, study of Rossoni et al. failed to include other than single reference strains of *C. albicans*. On the basis of single isolates, one must not drew any relevant conclusions.

Moreover, switching the sensitizer can totally reverse the observed response to photodynamic treatment [4]. Consequently, such a general conclusion proposed by the authors stating that *C. albicans* serotype B is more sensitive to photoinactivation is not supported by the evidence, as it requires studies concerning different sensitizing agents. However, the authors have not provided any details about the effectiveness of photoinactivation against both *C. albicans* serotypes based on other photosensitizers.

Assessing the influence of microorganism’s features on susceptibility to photoinactivation is always a complex issue. Of course, studying one sensitizer and single isolates can give us some information, but the problems in study design should not be ignored, as statistical analysis requires more numerous groups to be compared and to draw relevant conclusions. Those limitations reduce the reliability of presented results.
